# Cross‐site harmonization of diffusion MRI data without matched training subjects

**DOI:** 10.1002/mrm.30575

**Published:** 2025-05-23

**Authors:** Alberto De Luca, Tine Swartenbroekx, Harro Seelaar, John van Swieten, Suheyla Cetin Karayumak, Yogesh Rathi, Ofer Pasternak, Lize Jiskoot, Alexander Leemans

**Affiliations:** ^1^ Image Sciences Institute, Center for Image Sciences University Medical Center Utrecht Utrecht the Netherlands; ^2^ Department of Neurology and Alzheimer Center Erasmus MC University Medical Center Rotterdam the Netherlands; ^3^ Brigham and Women's Hospital Harvard Medical School Boston Massachusetts USA

**Keywords:** brain, diffusion mri, diffusion tensor imaging, harmonization

## Abstract

**Purpose:**

Diffusion MRI (dMRI) data typically suffer of significant cross‐site variability, which prevents naively performing pooled analyses. To attenuate cross‐site variability, harmonization methods such as the rotational invariant spherical harmonics (RISH) have been introduced to harmonize the dMRI data at the signal level. A common requirement of the RISH method is the availability of healthy individuals who are matched at the group level, which may not always be readily available, particularly retrospectively. In this work, we propose a framework to harmonize dMRI without matched training groups.

**Methods:**

Our framework learns harmonization features while controlling for potential covariates using a voxel‐based generalized linear model (GLM). RISH‐GLM allows us to simultaneously harmonize data from any number of sites while also accounting for covariates of interest, thus not requiring matched training subjects. Additionally, RISH‐GLM can harmonize data from multiple sites in a single step, whereas RISH is performed for each site independently.

**Results:**

We considered data of training subjects from retrospective cohorts acquired with three different scanners and performed three harmonization experiments of increasing complexity. First, we demonstrate that RISH‐GLM is equivalent to conventional RISH when trained with data of matched training subjects. Second, we demonstrate that RISH‐GLM can effectively learn harmonization with two groups of highly unmatched subjects. Third, we evaluate the ability of RISH‐GLM to simultaneously harmonize data from three different sites.

**Conclusion:**

RISH‐GLM can learn cross‐site harmonization both from matched and unmatched groups of training subjects and can effectively be used to harmonize data of multiple sites in one single step.

## 1 INTRODUCTION

Diffusion MRI (dMRI) has become a pivotal technique to investigate brain structure noninvasively.^1^ The signal measured with dMRI originates from the motion of water molecules at the microscopic scale.^2^ In combination with appropriate modeling,^3^ dMRI allows us to infer microstructural properties of tissues. For example, diffusion tensor imaging,^4^, ^5^ one of the most commonly applied dMRI techniques, provides metrics such as the mean diffusivity (MD) and the fractional anisotropy^4^, ^6^ (FA), which are related to the root mean square displacement and the anisotropy of the diffusion process, and reflect properties of the biological environment. Over time, these metrics have become established in the study of white‐matter (WM) microstructure and found application in the study of neurological diseases,^7^ including Alzheimer's disease,^8^ small vessel disease^9^, ^10^ and frontotemporal dementia,^11^ among others.

A possible limitation to the use of dMRI in clinical research is that its measurements strongly depend on the MRI hardware and software used.^12^, ^13^ As such, metrics derived from dMRI can typically be compared only within a single site, even when acquisition parameters are kept constant. In recent years, several methods have been proposed to tackle such cross‐site variability and harmonize dMRI. Broadly speaking, the goal of harmonization methods can be summarized as removing cross‐site differences while not altering the sensitivity of dMRI to biological effects of interest. Two main families of postprocessing dMRI harmonization methods have been proposed to date. The first family aims to remove batch effects during the analysis steps. Methods such as ComBat,^14^, ^15^, ^16^ for example, aim to remove cross‐site differences on the final metrics derived from dMRI. As such, harmonization is applied independently to each considered dMRI metric, such as FA or MD. The second family of dMRI harmonization methods aims to remove cross‐site effects on the acquired data before any quantification step. Notable examples hereof are the rotational invariant spherical harmonics^17^, ^18^ (RISH) framework, or other recently proposed deep learning alternatives.^19^, ^20^ Differently from methods such as ComBat, which harmonize end‐point diffusion metrics independently from each other, RISH and comparable frameworks harmonize the source dMRI data. As such, the source data are harmonized once as part of the preprocessing pipeline and can be then re‐used for multiple analyses with any method of choice. For example, we have previously demonstrated that dMRI data of patients with small vessel disease^21^, ^22^ can effectively be harmonized with RISH and then be used for both voxel‐wise‐based analyses and connectomics.

One of the challenges when applying dMRI harmonization methods such as RISH is that they require data of healthy participants for calibration between sites. For example, previous work^17^ has demonstrated that the RISH method requires 12 or more training subjects matched at the group level for each site to be harmonized, or repeated scans of so‐called traveling heads. This can be particularly challenging to achieve for studies focusing on patient populations, in whom healthy controls may not always be recruited, or when retrospectively harmonizing multiple different cohorts with differences in average age or sex distribution. Furthermore, the concept of matched groups is ill‐defined and study‐dependent. In practice, it is very often interpreted as a synonym of age and sex matching, but other factors such as education and the presence of genetic mutations, among others, may affect the dMRI signal and might need to be considered as part of the group matching for specific studies.

In this study, we propose a generalization of the RISH framework that determines cross‐site harmonization while also considering potential confounders. This new approach accounts for potential biases when learning harmonization from unmatched groups of training subjects, and allows us to harmonize data from multiple sites in one single step. We demonstrate the potential of this framework through three experiments of growing complexity using dMRI data of subjects from three different sites, while controlling for sex and age.

## 2 METHODS

### 2.1 Theory: RISH and RISH general linear model

The conventional RISH harmonization method is based on the representation of diffusion MRI data acquired at a certain b‐value (i.e., a diffusion “shell”) with spherical harmonics. Spherical harmonics are a set of functions on the unit sphere characterized by an order and a degree. For each order *i*, rotational invariant representations (i.e., RISH coefficients) can be determined and are indicated as $L_{i}$. Mirzaalian et al. and subsequent works^17^, ^23^ have shown that it is possible to harmonize dMRI data by learning the scaling factors between corresponding RISH coefficients determined in two groups of matched training subjects. The same framework can also be used to harmonize multishell data effectively by harmonizing each shell independently.^18^ The harmonization essentially determines a voxel‐wise scaling factor associated with each RISH feature to map a target site to a reference site. Mathematically, the scaling coefficient ϑ associated with the spherical harmonics of order *i* can be written as follows:

(1) ϑi=σi,Rσi,T

where σ indicates the average RISH features of the reference (R) and target sites (T). Subsequently, dMRI data sets from the target site can be harmonized as follows: (i) dMRI data are converted to spherical harmonics; (ii) their RISH features are calculated and multiplied by the scaling factor $\vartheta_{i}$; and (iii) harmonized dMRI data are reconstructed from the corrected spherical harmonics representation. The assumption behind this approach is that cross‐site differences in σ purely originate from scanner‐related properties. For this assumption to hold, the groups from which the σ values are computed must be matched across all factors that may affect the dMRI signal. In practice, matching for all relevant factors can be challenging. When this is not the case, residual systematic group differences will be assimilated into the scaling factors, potentially biasing the factors and making them less generalizable to harmonize other subjects from the same site.

To alleviate the matching requirement, we propose a new formulation based on a general linear model (GLM) that decomposes the effect of confounders such as age and sex on the RISH features, as follows:

(2) $$\left[\begin{array}{c} \sigma_{i,R}\\ \sigma_{i,T} \end{array}\right]=\left[S_{R}\;S_{T}\;\mathrm{COV}\right]\left[\begin{array}{c} \beta_{i,R}\\ \beta_{i,T}\\ \beta_{i,\mathrm{COV}} \end{array}\right]$$

where S are dummy variables assuming a value of 1 when a subject belongs to a certain site, and 0 otherwise. This approach, dubbed RISH‐GLM, is based on two assumptions: (i) that the relationship between RISH features and covariates is linear in the considered range; and (ii) limited collinearity and interaction between different covariates. Of note, linearity in the relation between covariates and RISH‐features does not imply nor require linearity between covariates and diffusion metrics (e.g., FA). The RISH‐GLM formulation allows us to alleviate the need to match the average demographics at the group level across all sites. In practice, however, a reasonably even distribution of the effects of interest across sites is still needed to reliably estimate the associated coefficients β, from which the scaling coefficients can be determined as ϑi=βi,1βi,2. This formulation can readily be extended to account for multiple sites by considering additional columns on the right side as follows:

(3) $$\left[\begin{array}{c} \sigma_{i,R}\\ \sigma_{i,T1}\\ \vdots \end{array}\right]=\left[\begin{array}{ccc} S_{R} & S_{T1}\;\cdots & \mathrm{COV} \end{array}\right]\left[\begin{array}{c} \beta_{i,R}\\ \beta_{i,T1}\\ \vdots \\ \beta_{i,COV} \end{array}\right]$$

### 2.2 MRI data

We retrospectively collected data from the Frontotemporal Dementia Risk Cohort^24^ (FTD‐RISC), which is a longitudinal study following individuals at risk of frontotemporal dementia (FTD) curated at the Erasmus MC University Medical Center in Rotterdam, the Netherlands. In FTD‐RISC, three major combinations of MRI hardware and software have been used, which we refer to as Site 1, Site 2, and Site 3. Data from all sites are used throughout this study to demonstrate the potential of GLM‐RISH. T_1_‐weighted MRI data were acquired at all sites with a resolution of 1 × 1 × 1 mm^3^ isotropic. dMRI data were acquired at all sites with a similar dMRI protocol featuring one *b* = 0 s/mm^2^ volume and 60 gradient directions at *b* = 1000 s/mm^2^. Data of Site 1 were acquired with a 3T Philips Achieva scanner located at Leiden University Medical Center (the Netherlands) running on software release R5 with a 32‐channel head coil. At this site, key imaging parameters were voxel size = 2 × 2 × 2 mm^3^, echo time (TE) = 80 ms, and repetition time (TR) = 9.25 s. Data of Site 2 were acquired with a GE Healthcare MR750 3T scanner located at Erasmus Medical Center (the Netherlands) with a 48‐channel head coil. At this site, key imaging parameters were voxel size = 2 × 2 × 2 mm^3^, TE = 57 ms, and TR = 9 s. Data of Site 3 were acquired with an older version of the scanner at Site 1, running on software release R4 and equipped with an 8‐channel head coil. At this site, dMRI data were acquired with voxel size = 2 × 2 × 2 mm^3^, TE = 80 ms, and TR = 8.25 s. For this study, only data of healthy control subjects (noncarriers) and pathogenic variant carriers who did not have FTD‐related symptoms at the time of scanning were considered.

### 2.3 MRI processing

MRI data were processed with a previously presented automated processing pipeline.^25^ T_1_‐weighted data were processed with *CAT12* (https://neuro‐jena.github.io/cat/) to remove bias fields and to perform skull stripping. dMRI data were processed with *MRIToolkit* (https://github.com/delucaal/MRIToolkit) and *ExploreDTI* ^26^, ^27^ to perform signal drift correction,^28^ then denoised with the Marchenko‐Pasteur principal component analysis method.^29^ Subsequently, corrections for Gibbs ringing,^30^ motion, and echo‐planar imaging corrections were performed, including b‐matrix rotation.^31^ Echo‐planar imaging distortions were corrected by means of a nonlinear registration to the T_1_‐weighted data at a 2‐mm resolution. Robust estimation of the diffusion tensor was performed using *REKINDLE*,^32^ then the FA and the MD were calculated.^5^ Quality assessment of the data was performed by generating summary screenshots of FA, MD, and fit residuals of each data set, which were visually inspected by a trained researcher (ADL). Data exhibiting excessive motion or apparent artifacts (e.g., due to braces or other interference sources) were discarded.

### 2.4 Harmonization experiments

We refer to RISH[Matched/Unmatched] and RISH‐GLM[Matched/Unmatched] to indicate whether the methods were trained with matched or unmatched groups of participants from different sites, respectively. To perform RISH and RISH‐GLM harmonization, spherical harmonics were fitted to dMRI data using in‐house software written in *MATLAB*, which implemented L2 regularized least squares. RISH features were then calculated and spatially normalized to a study specific template as previously proposed.^17^ The code used to perform the aforementioned steps is openly available at https://github.com/delucaal/RISH‐GLM.

We evaluated the performance of RISH‐GLM as compared to conventional RISH with three experiments of growing complexity. Table 1 provides an overview of the data sets used in the three experiments.

**TABLE 1:** Overview of the data sets used to train RISH and RISH‐GLM in the three experiments.

Data sets	*N* (per site)	Age (years)	Sex (F)
Data Set 1	15 (Site 1) vs. 15 (Site 2)	50.2 ± 12.2 vs. 52.8 ± 11.1	47% vs. 33%
Data Set 2	18 (Site 1) vs. 18 (Site 2)	35.4 ± 6.1 vs. 56.4 ± 8.5	44% vs. 39%
Data Set 3	15 (Site 1) vs. 15 (Site 2) vs. 15 (Site 3)	48.4 ± 10.2 vs. 52.8 ± 10.8 vs. 56.5 ± 8.9	40% vs. 33% vs. 53%
Data Set 4	15 (Site 1) vs. 15 (Site 2) vs. 15 (Site 3)	52.3 ± 7.5 vs. 55.5 ± 10.1 vs. 50.5 ± 7.6	40% vs. 40% vs. 40%

*Note*: *N* = sample size of each site. F = female.

Abbreviations: GLM, generalized linear model; RISH, rotational invariant spherical harmonics.

In Experiment 1, we investigate whether RISH‐GLM is equivalent to the conventional RISH method when trained on data of 15 individuals matched at the group level for both age and sex from Data Set 1. For each spherical harmonics's order, the scaling factors were computed with both RISH and RISH‐GLM, accounting for both age and sex, and visually compared. Subsequently, the training data from Site 2 were harmonized, and the diffusion tensor model was fit^32^ as mentioned in Section 2.3. FA maps were computed and registered to a common space using the TBSS_PNL pipeline (https://github.com/pnlbwh/tbss). In short, this pipeline is based on the FSL TBSS^33^ approach but replaces the registration steps with ANTS^34^ for additional spatial accuracy. Boxplots of FA computed on the WM skeleton were compared before and after harmonization. Given that FA is known to be strongly associated with age,^35^ we additionally visualized their relation by means of scatterplots and evaluated their Pearson correlation coefficient before and after harmonization. As no ground truth exists for age and sex effects, we evaluated the agreement between the effects estimated by RISH‐GLM on the whole data sets, and age and sex effects within the individual sites by means of Pearson correlations. To estimate the effects within the individual sites, GLMs accounting for age, sex, and intercept were estimated at the voxel level for Site 1 and Site 2 independently.

Subsequently, in Experiment 2, we evaluated the feasibility of removing scanner‐related cross‐site differences by learning harmonization with two age‐unmatched groups of subjects from Data Set 2. RISH‐GLM was trained by providing age and sex of each individual as covariates. As in the previous experiment, we compared the scaling factors corresponding to different RISH features and visually inspected the spatial maps corresponding to age and sex effects. Harmonization was applied to the training set (Data Set 2). Boxplots of FA and scatterplots of FA as a function of age were derived as explained in Experiment 1. Considering the large age difference between the two groups in Data Set 2, we anticipated observing differences in FA between the two groups before harmonization. We expected such differences not to be completely removed after harmonization if the applied method truly removes only scanner‐related differences and not biological effects. Furthermore, we expected a successful harmonization to recover a Pearson correlation coefficient between age and FA as similar as possible to the one determined in Experiment 1. The same analyses were repeated on an independent data set (Data Set 1) for validation purposes. We anticipated that RISH‐GLM harmonization trained with age‐unmatched subjects (Data Set 2) would generalize to age‐matched subjects (Data Set 1), whereas RISH harmonization would not.

In Experiment 3, we evaluated whether the RISH‐GLM framework allows us to effectively harmonize data from multiple sites from Data Set 3 in a single step. FA maps were calculated and aligned to a common space as previously explained. Next to boxplots of FA derived on the WM skeleton, we additionally performed voxel‐wise t‐tests while correcting for age and sex to also evaluate the effectiveness of RISH‐GLM at removing potential regional cross‐site differences. Comparisons among the three sites were performed pairwise (i.e., Site 1 vs. Site 2 and Site 3, Site 2 vs. Site 3) using FSL *randomize* with threshold‐free cluster enhancement. Finally, to validate the one‐step RISH‐GLM harmonization on independent subjects, we applied the trained harmonization to age‐matched and sex‐matched subjects of Data Set 4, which shared only 2 out of 45 subjects with Data Set 3. Here we evaluated the effectiveness of RISH‐GLM at removing cross‐site differences by means of boxplots of FA calculated on the WM skeleton, and by evaluating the relation between FA and age as in the previous experiments.

In all experiments, Site 1 was used as reference, and its values should remain constant. However, boxplots of the reference site might exhibit slight differences because of how the TBSS_PNL pipeline—used to compute skeletonized values for the boxplots—inherently works. TBSS_PNL constructs a WM skeleton based on all input data (both reference and target sites). Because harmonization alters the target site's data, the global skeleton changes slightly, which can introduce minor shifts in the reference site's FA distributions.

## 3 RESULTS

### 3.1 Experiment 1: RISH‐GLM and RISH with matched training subjects (expect no differences)

**FIGURE 1:**
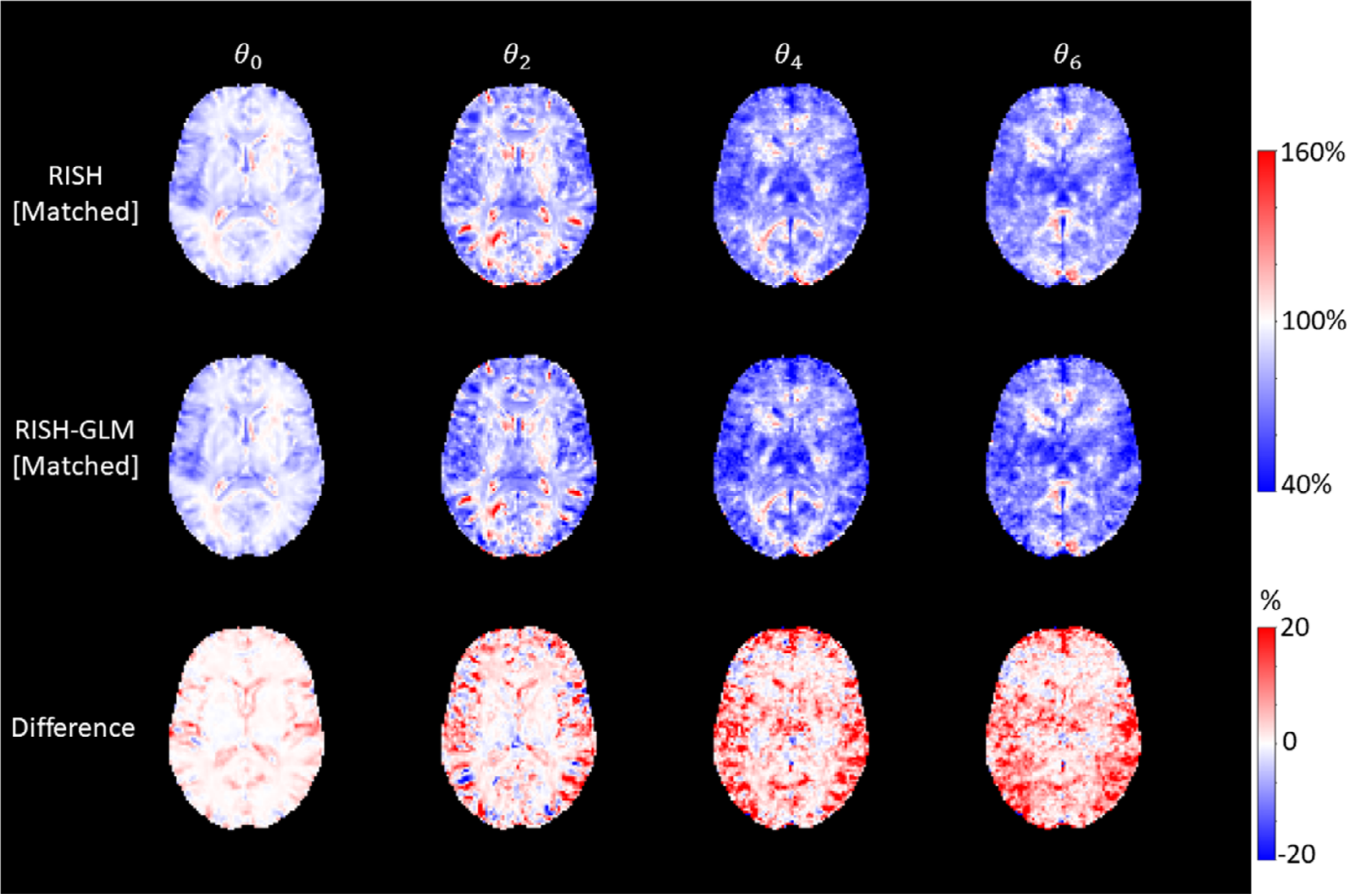
Voxel‐wise scaling maps calculated in Data Set 1 using group level‐matched training subjects with rotational invariant spherical harmonics (RISH) (*first row*), rotational invariant spherical harmonics–based generalized linear model (RISH‐GLM) (*second row*), and their difference (*last row*) for different orders of spherical harmonics (*columns*). Scaling maps calculated from both methods are globally similar, but local differences up to ±20% can be noticed particularly at the interface between gray matter and cerebrospinal fluid, and in the deep white matter for $\theta_{4}$ and $\theta_{6}$.

Figure 1 shows the scaling maps $\vartheta_{i}$ corresponding to spherical harmonics up to order 6 that were calculated with RISH and RISH‐GLM using training subjects matched for age and sex at the group level from two different sites (hence the “[Matched]” suffix). The maps calculated by both methods are remarkably similar, as shown by the corresponding relative difference maps. The average relative differences between RISH[Matched] and RISH‐GLM[Matched] are 0.38% for $\vartheta_{0}$, 0.73% for $\vartheta_{2}$, 1.32% for $\vartheta_{4}$, and 1.45% for $\vartheta_{6}$. Although differences are globally less than 2%, local differences up to 20% can be appreciated in the bottom row of Figure 1. Such differences are mostly located in areas of partial volume with cerebrospinal fluid (CSF) or gray matter (GM), but not in deep WM, and might originate from small imbalances in covariates or site‐specific artifacts. Indeed, age and sex maps estimated with RISH‐GLM (Figure S1) show that these two covariates can have a small but nonzero spatially varying effect on RISH scales, even when these are calculated in two matched groups.

In Data Set 1, age and sex effects estimated with RISH‐GLM in WM are generally in agreement with age effects calculated within Site 1 and Site 2 independently. The correlation coefficient between age effects estimated in the whole cohort and in Sites 1/2 are 0.80/0.74 for $\vartheta_{0}$, 0.86/0.87 for $\vartheta_{2}$, 0.62/0.83 for $\vartheta_{4}$, and 0.55/0.83 for $\vartheta_{6}$, respectively. The correlation coefficient for sex effects estimated in the whole cohort and in Sites 1/2 are 0.75/0.70 for $\vartheta_{0}$, 0.72/0.70 for $\vartheta_{2}$, 0.53/0.89 for $\vartheta_{4}$, and 0.56/0.89 for $\vartheta_{6}$, respectively.

**FIGURE 2:**
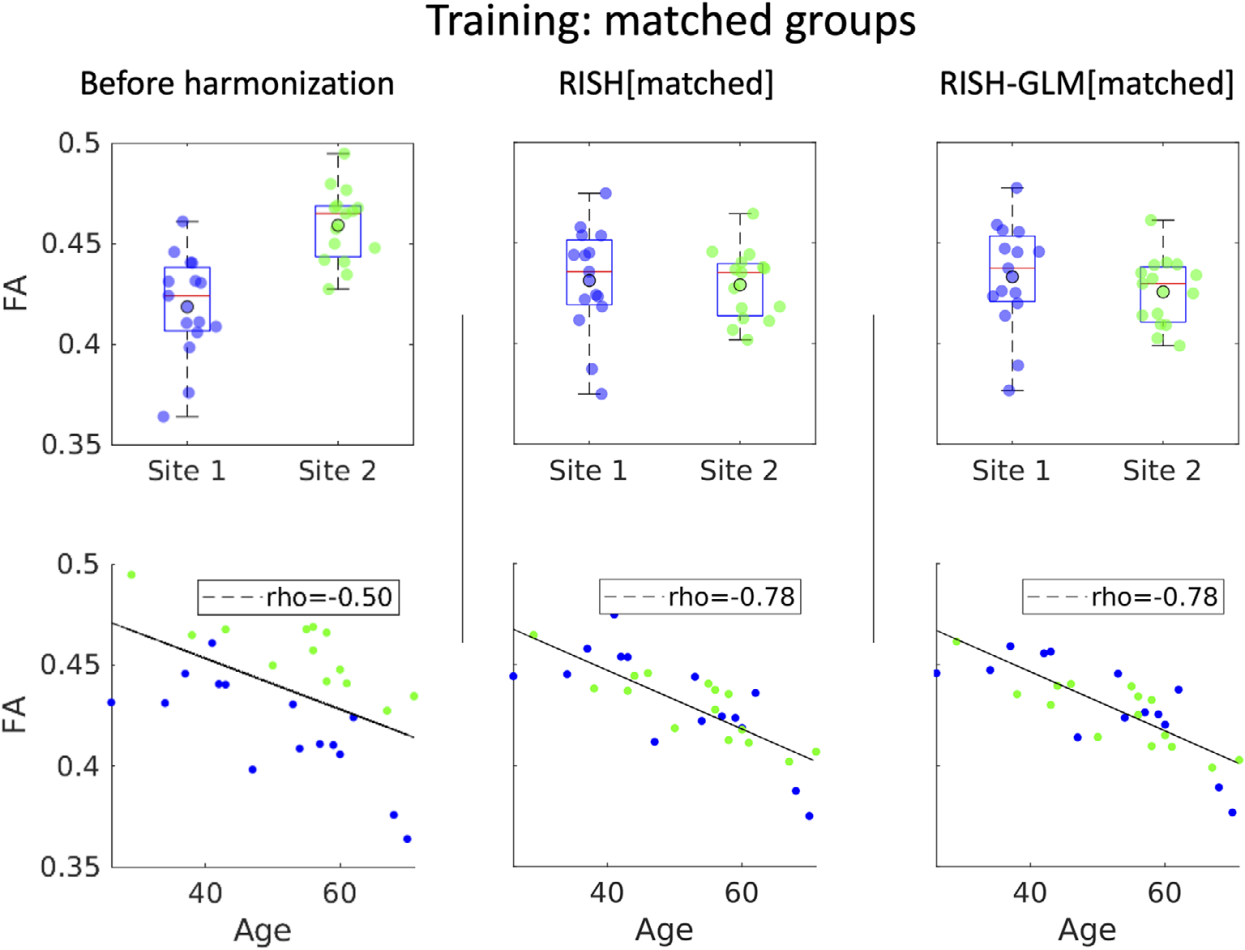
The top row shows boxplots of average fractional anisotropy (FA) values in the white‐matter skeleton per site of Data Set 1, before harmonization (*first column*), after harmonization with rotational invariant spherical harmonics (RISH) (*middle column*), and with rotational invariant spherical harmonics–based generalized linear model (RISH‐GLM) (*last column*). The second row shows scatterplots of the same average FA values as a function of age. Harmonization with both RISH and RISH‐GLM was trained with group level‐matched subjects.

Figure 2 shows boxplots of average FA values of Site 1 and Site 2 obtained on the WM skeleton before harmonization, and after harmonization with RISH and RISH‐GLM. Before harmonization, a relative difference in average FA values between Site 1 and Site 2 equal to −9.7% (significant t‐test, *p* ≤ 0.05) can be observed, which is effectively removed by both RISH (0.5%, *p* = 0.80) and RISH‐GLM (1.7%, *p* = 0.37). The second row of Figure 2 shows the existence of a negative relation between age and FA within individual sites. When pooling unharmonized data together (first row), a biased correlation coefficient (rho) equal to −0.5 can be observed. After harmonization with both RISH and RISH‐GLM, a linear negative relation between age and FA can be observed with equal correlation coefficients rho = −0.78. In both cases, the measurements from each site are well distributed around the regression line.

### 3.2 Experiment 2: RISH‐GLM and RISH with unmatched training subjects

**FIGURE 3:**
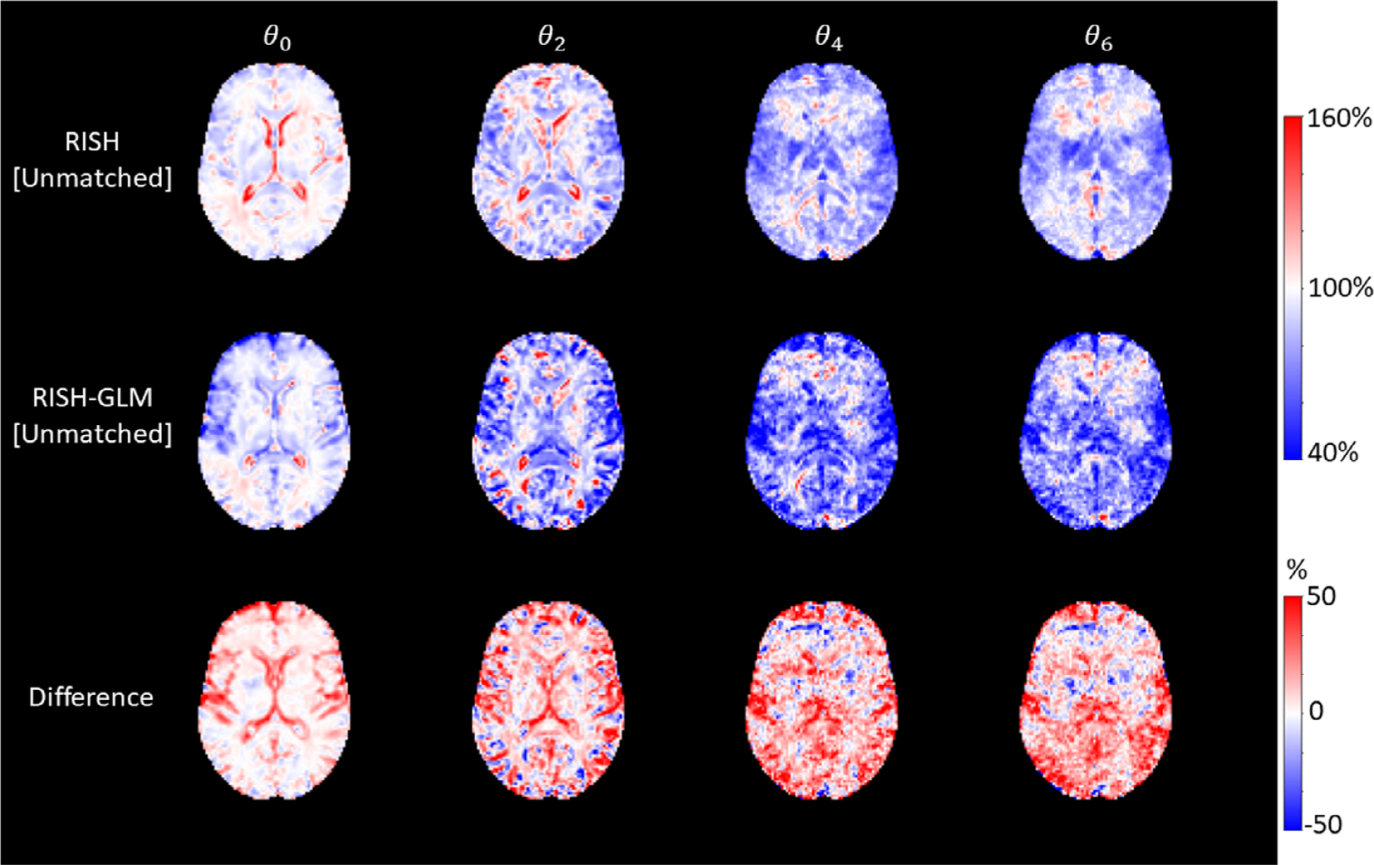
Voxel‐wise scaling maps calculated using group age‐unmatched training subjects (Data Set 2) with rotational invariant spherical harmonics (RISH) (*first row*), rotational invariant spherical harmonics–based generalized linear model (RISH‐GLM) (*second row*), and their difference (*last row*) for different orders of spherical harmonics (*columns*). Compared with Figure 1 (matched training groups), scaling maps calculated show different patterns with local differences up to ±50%.

Figure 3 shows the scaling maps calculated for different orders of spherical harmonics with RISH and RISH‐GLM trained with two groups of subjects that were not matched at the group level (hence the suffix “[Unmatched]”). In contrast to Figure 1, where RISH and RISH‐GLM produced similar scaling maps, large differences between the two methods can be observed when using unmatched training subjects, particularly for higher orders of spherical harmonics. Compared with the reference scaling maps calculated with RISH with matched subjects (Figure 1), scaling maps calculated with unmatched subjects with RISH show large differences particularly around the ventricles, and at the interface between GM and CSF for $\vartheta_{0}$ and $\vartheta_{2}$. For higher spherical harmonics orders (i.e., $\vartheta_{4}$ and $\vartheta_{6}$), widespread differences in both the WM and the GM can be observed. Compared with the reference (RISH[Matched]), average relative differences of RISH[Unmatched] and RISH‐GLM[Unmatched] are −10.2% and −3.3% for $\vartheta_{0}$, −12.0% and −2.6% for $\vartheta_{2}$, −10.5% and 2.9% for $\vartheta_{4}$, and −10.6% and 3.1% for $\vartheta_{6}$, respectively, highlighting that scaling maps computed with RISH‐GLM[Unmatched] are on average closer to the reference (RISH[Matched]) than RISH[Unmatched].

**FIGURE 4:**
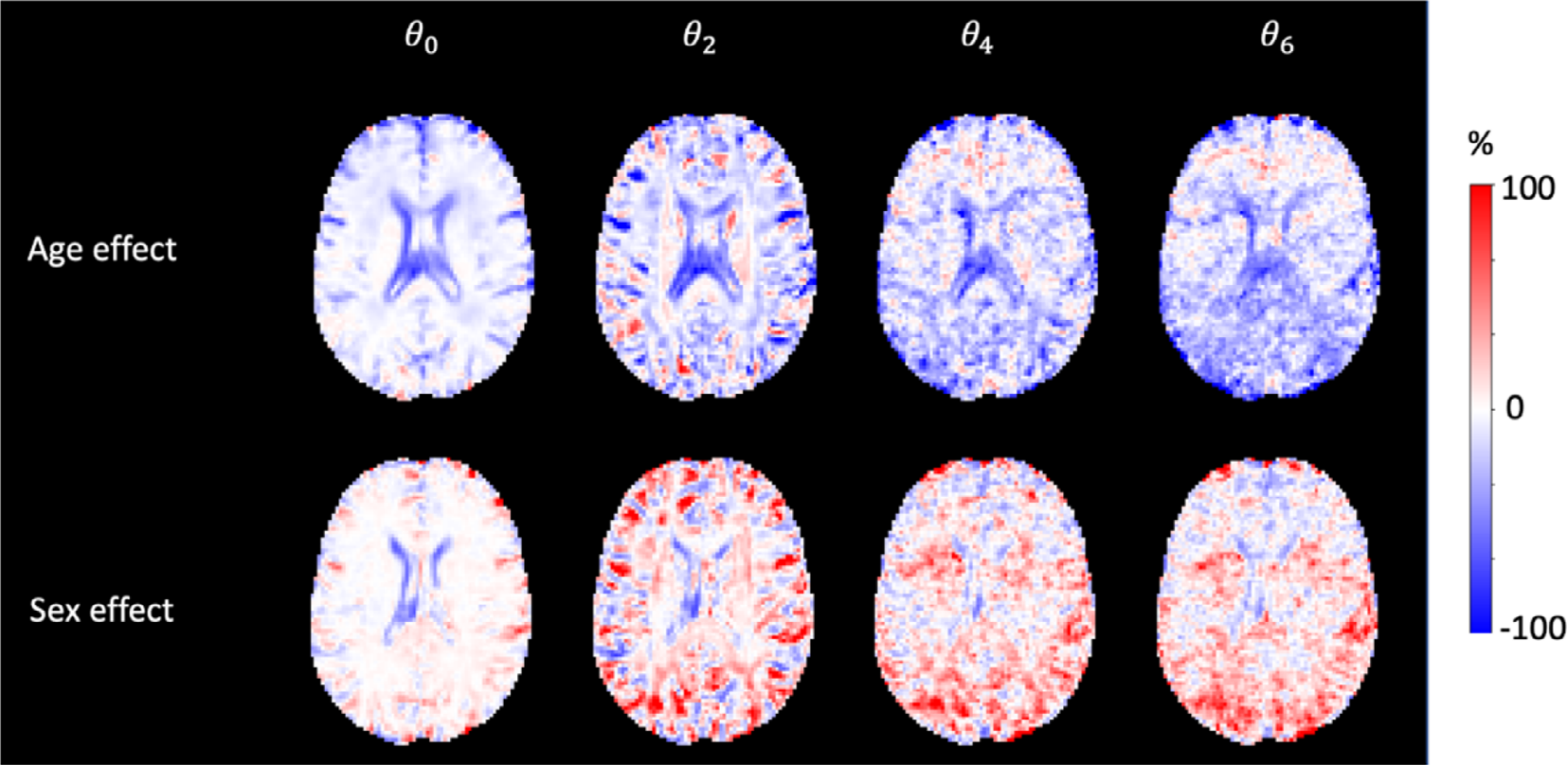
Percentage effect of age and sex estimated by rotational invariant spherical harmonics–based generalized linear model (RISH‐GLM)[Unmatched] in Data Set 2. Age effects are prevalent at the interface between cerebrospinal fluid and white/gray matter for $\vartheta_{0}$, and in the white matter for $\vartheta_{2}$ and $\vartheta_{4}$. The age effect map for $\vartheta_{6}$ appears to be dominated by noise effects, given the low anatomical contrast, and the presence of clear stripes due to ghosting artifacts. Similar observations hold for sex effects.

Figure 4 shows the spatial coefficients of age and sex effects associated with different spherical harmonics orders. Considering $\vartheta_{0}$, it can be appreciated that age has a large effect around the ventricles and at the interface between GM and CSF. At higher orders, age has a stronger effect on the brain WM, particularly in the corpus callosum and corticospinal tracts. Similar observations also hold for sex effects. Importantly, both covariates have the largest effects in areas where RISH[Matched] and RISH‐GLM[Unmatched] differ the most. In Data Set 2, age and sex effects estimated with RISH‐GLM in WM are generally in agreement with age effects calculated within groups of Data Set 2 independently. The correlation coefficient between age effects estimated in the whole cohort and in Sites 1/2 are 0.58/0.80 for $\vartheta_{0}$, 0.56/0.80 for $\vartheta_{2}$, 0.25/0.90 for $\vartheta_{4}$, and 0.26/0.89 for $\vartheta_{6}$, respectively. The correlation coefficient for sex effects estimated in the whole cohort and in Sites 1/2 are 0.66/0.68 for $\vartheta_{0}$, 0.69/0.64 for $\vartheta_{2}$, 0.50/0.81 for $\vartheta_{4}$, and 0.51/0.80 for $\vartheta_{6}$, respectively.

**FIGURE 5:**
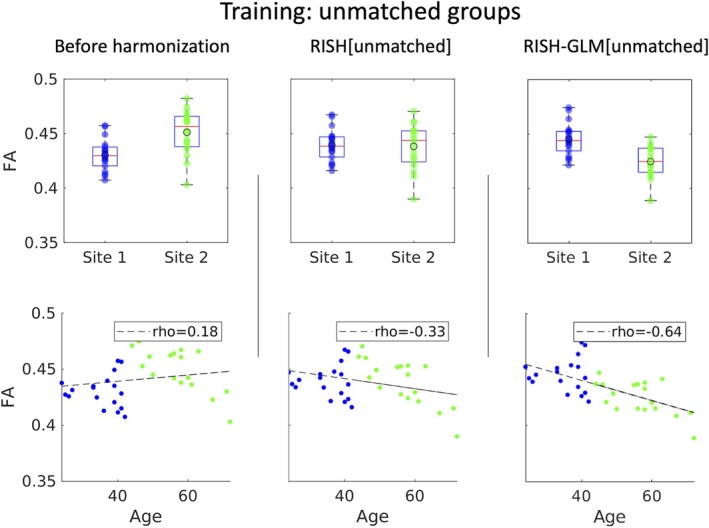
Boxplots of average fractional anisotropy (FA) values from two unmatched groups of healthy controls from Site 1 and Site 2 (Data Set 2), before harmonization, and after harmonization with rotational invariant spherical harmonics (RISH) and rotational invariant spherical harmonics–based generalized linear model (RISH‐GLM). Before harmonization, an average difference in FA between the two groups can be observed. Although this could be plausible given the unmatched nature of the two groups, there is an unexpected lack of correlation between age and FA before harmonization. Harmonization with RISH[Unmatched] removes the difference between the two groups. Consequently, a mild negative correlation between age and FA is observed, but the datapoints from both sites are not well distributed around the regression line, indicating a biased fit. After harmonization with RISH‐GLM[Unmatched], a difference between the two groups in average FA values is observed, as expected, as well as a clear negative relation between age and FA.

In Figure 5, we evaluated the average FA values calculated in the WM skeleton of the unmatched groups. Before harmonization, differences in average FA values equal to −4.8% (*p* ≤ 0.05) between the two sites can be observed. Importantly, such differences can originate from both scanner‐specific differences as well as from true biological effects given the large age span. Before harmonization, no correlation is observed between age and FA. In this situation, applying RISH[Unmatched] while ignoring age and sex effects reduces the average FA difference between the two groups to 0.1% (*p* = 0.95) but suppresses the expected difference between two groups with largely different age distributions.^35^, ^36^ After harmonization with RISH‐GLM[Unmatched], a relative difference in average FA values equal to 4.6% can be observed (*p* ≤ 0.05), together with a linear negative relation between age and average FA that is in line with what was observed in Figure 2 (correlation coefficient rho = −0.64 vs. −0.78 in Figure 2). Similar considerations hold also for MD, as shown in Figure S2.

**FIGURE 6:**
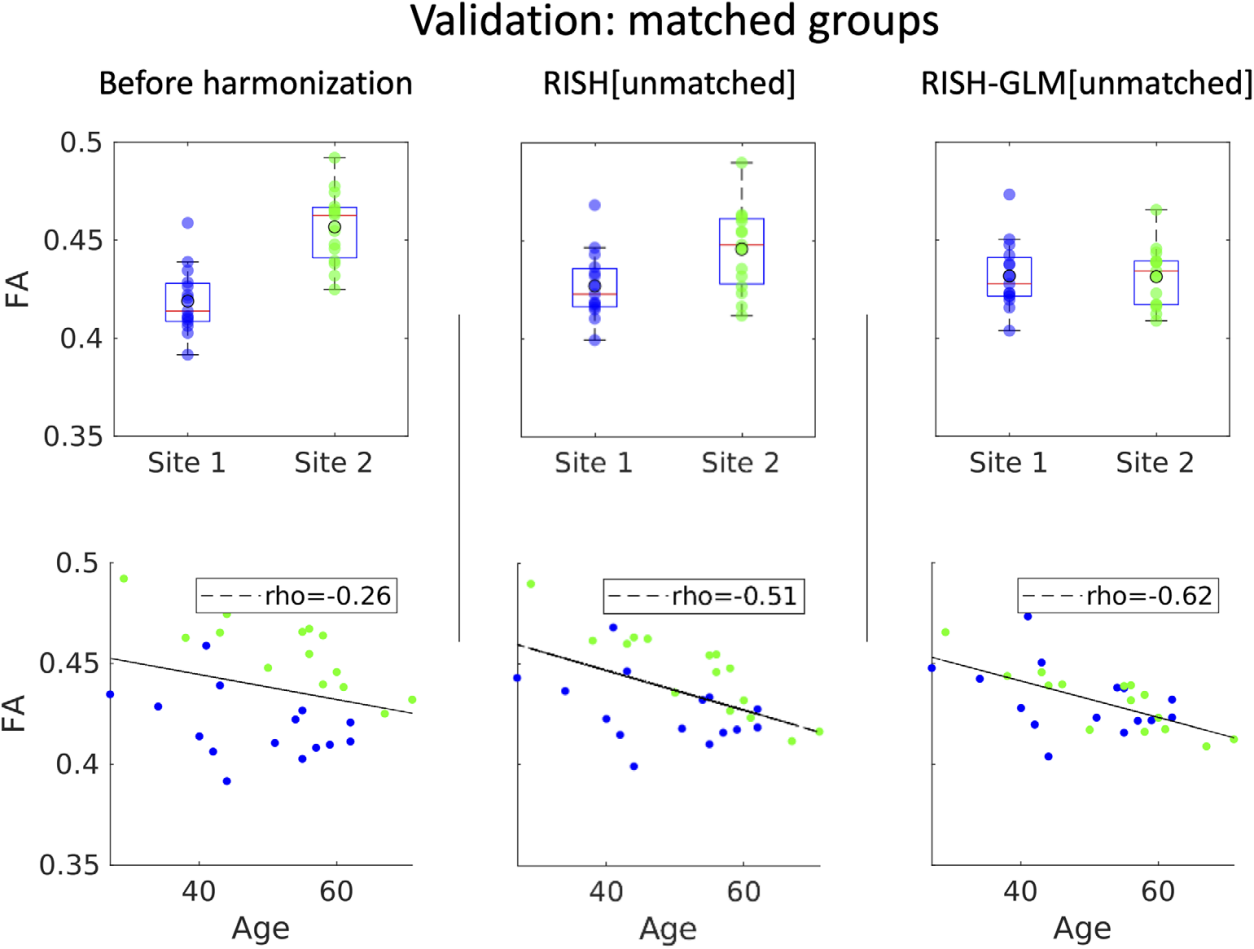
Boxplots of average fractional anisotropy (FA) values from two matched groups from Site 1 and Site 2 (Data Set 2) before harmonization, and after applying rotational invariant spherical harmonics (RISH) and rotational invariant spherical harmonics‐based generalized linear model (RISH‐GLM) trained on unmatched data (Figure 5). No differences in average FA values are observed after harmonization with RISH‐GLM, as expected for two matched groups. The application of RISH‐GLM also allows to reveal the same negative correlation between age and FA observed in the previous figures.

Subsequently, we have applied RISH[Unmatched] and RISH‐GLM[Unmatched] to harmonize the data of the two matched groups of Data Set 1, for which no average difference in FA values is expected after harmonization. The boxplots shown in Figure 6 showcase how RISH‐GLM[Unmatched] can effectively remove the cross‐site differences in FA between the two groups (average relative difference 0.5%, *p* = 0.71), whereas RISH[Unmatched] does not (average relative difference = −4.4%, *p* ≤ 0.05). When looking at the relation between age and FA, the application of RISH‐GLM[Unmatched] recovers the same age–FA relation observed also in Figures 2 and 5. Similarly, RISH‐GLM[Unmatched] effectively also harmonizes MD, as shown in Figure S3, whereas RISH[Unmatched] does not.

### 3.3 Experiment 3: One‐step harmonization

**FIGURE 7:**
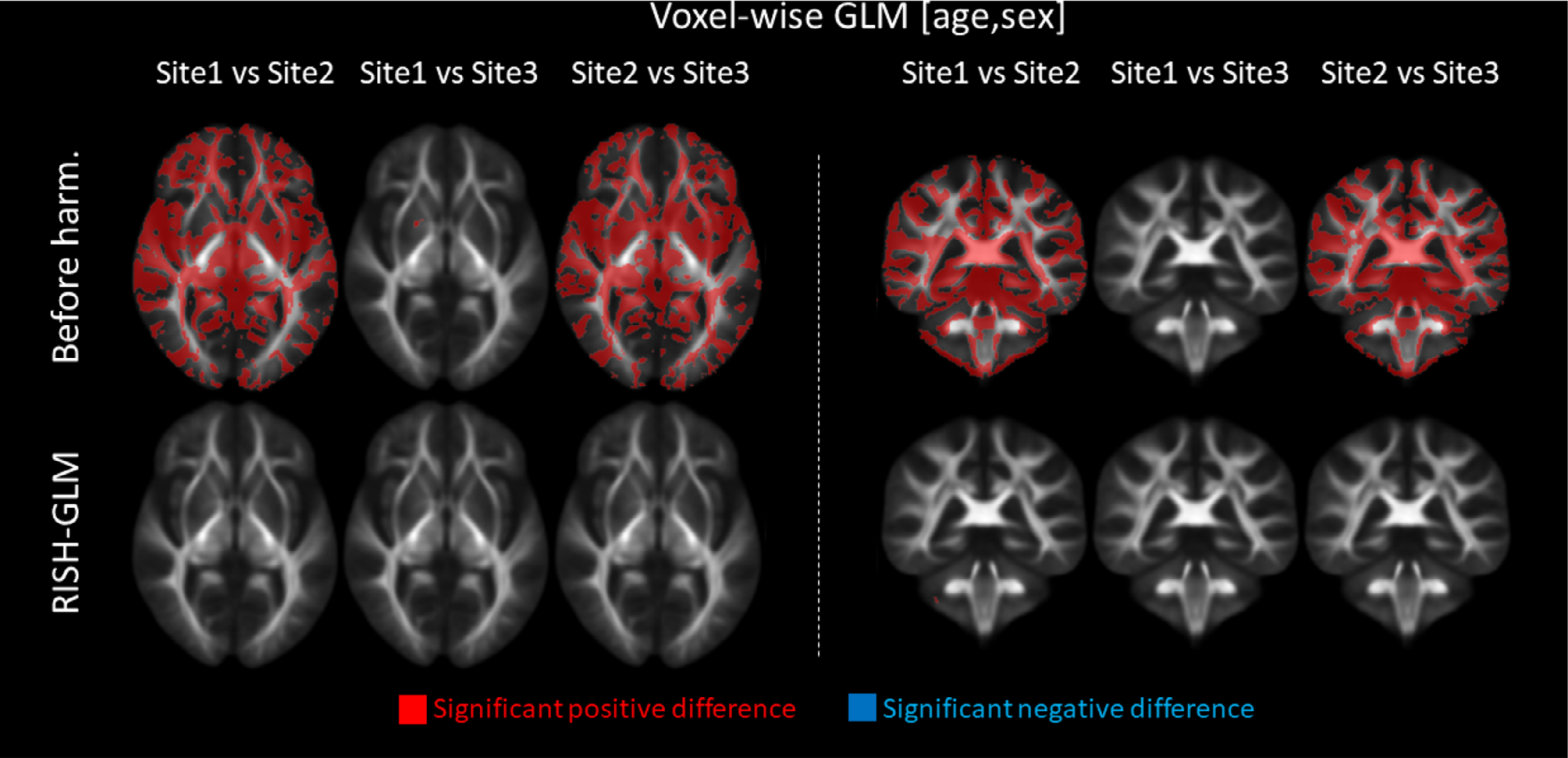
Example axial (*left*) and coronal (*right*) slices showing differences in average fractional anisotropy (FA) values at the voxel level between pairs of sites of Data Set 3 by applying a permutation test corrected for age and sex with a general linear model to three unmatched groups. Before harmonization, minimal to no differences are observed between Site 1 and Site 3. Conversely, extensive differences are observed between Site 2 and the other sites. After harmonization with rotational invariant spherical harmonics–based generalized linear model (RISH‐GLM) in a single step, most differences are effectively removed.

We investigated the ability of RISH‐GLM to harmonize data from three sites in a single step. Example axial slices of the training scales derived with RISH and RISH‐GLM between corresponding pairs of sites are shown in Figure S4. Scaling maps computed from both methods have similar appearance; it can be appreciated how the scaling computed with RISH‐GLM are higher on average than those computed with RISH. The presence of ghosting artifacts can be appreciated in the scaling maps $\vartheta_{4}$ and $\vartheta_{6}$, particularly with RISH‐GLM. For this method, systematic artifacts can be learned as part of cross‐site differences and can propagate to age and sex effect maps, as shown in Figure S5. We subsequently evaluated differences in voxel‐wise FA values between pairs of sites by means of permutations tests corrected by age and sex, obtaining the maps shown in Figure 7. Before harmonization, 32.1%, 0.25%, and 29.6% of the brain voxels were statistically different when comparing Site 1 to Site 2, Site 1 to Site 3, and Site 2 to Site 3, respectively. After harmonization, the amount of significantly different voxels was reduced to 0.1%, 0.1%, and 0.4%, respectively. Similar observations apply to MD, for which significant differences were observed in respectively 25.6%, 0%, and 31.6% of brain voxels when comparing sites in the same pairwise as previously reported. After harmonization, significant differences were reduced to 0.1%, 0%, and 1.2% of brain voxels, respectively.

**FIGURE 8:**
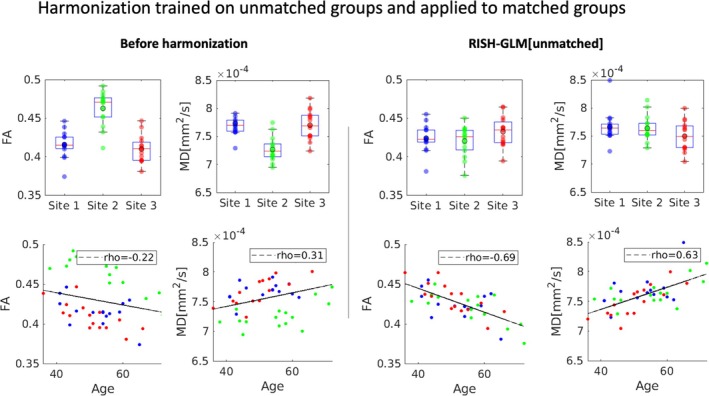
A comparison of average fractional anisotropy (FA) and mean diffusivity (MD) values calculated in the white‐matter skeleton for three matched groups of subjects from Site 1, Site 2, and Site 3 (Data Set 4) before and after harmonization in a single step with rotational invariant spherical harmonics–based generalized linear model (RISH‐GLM). After harmonization, no statistically significant difference among the three sites can be observed in both FA and MD, and values are well distributed around the regression line in the correlation plot between age and FA for all sites.

To validate the method, we have applied RISH‐GLM to harmonize the independent Data Set 4. Results, which are reported in Figure 8, demonstrate that RISH‐GLM can effectively reduce the differences among all cohorts, and recover the same relation between age and FA/MD previously observed in Figures 2, 5, and S6.

## 4 DISCUSSION

In this work, we have introduced a novel approach to perform RISH‐based harmonization of the dMRI signal without need for training subjects matched at the group level. Our approach does so by learning the voxel‐wise effects of covariates on RISH scaling coefficients with a GLM (RISH‐GLM). Our results demonstrate that RISH‐GLM is effective in learning cross‐site harmonization from unmatched training groups and can be used to harmonize data from multiple sites effectively in a single step.

The advent of dMRI harmonization methods such as RISH allows studies to overcome the barriers of single‐site analyses, unharnessing the potential of retrospectively collected data to boost sample sizes. However, the applicability of such frameworks can be challenging when considering multiple cohorts selected with different inclusion criteria because of their requirement of matched training. Importantly, the results shown in Figures 5 and 6 demonstrate that conventional RISH is unsuitable to learn cross‐site harmonization with unmatched groups, as that would lead to a removal of both cross‐site and biological effects (e.g., aging) (Figure 5), or to the introduction of biases (Figure 6). RISH‐GLM can effectively tackle this limitation by lifting the need for matched training groups, as demonstrated in Figures 6 and 8.

The ability to learn cross‐site harmonization without matched cohorts could support several efforts in both clinical and neuroscientific research. First, RISH‐GLM could simplify the harmonization of cohorts with only partly overlapping covariates of interest. This could be, for example, age, in the case of studies pooling retrospective cohorts with distinct age ranges to cover the whole lifespan. Second, it could support further disentangling scanner effects from confounding biological effects than conventional RISH, particularly when learning harmonization from groups that are matched on average but feature relevant variance in covariates. In the study of dementia, for example, population cohorts, memory clinic cohorts, and population with different injury etiologies could be potentially harmonized while accounting for key factors that could otherwise bias the harmonization process, such as differences in education, ethnicity, sex distributions, exposure to specific risk factors, lesion burden, and so forth.

Although retrospective data are arguably the main application goal of postprocessing dMRI harmonization methods such as RISH‐GLM, they could prove advantageous also to support prospective studies. In general, accurate matching of acquisition hardware and sequence parameters can already reduce cross‐site dMRI variability, as shown by previous initiatives in frontotemporal^37^ and vascular dementia.^38^ Yet, subtle differences among vendors, calibration, and characteristics of individual scanners might still dictate the existence of cross‐site differences. Furthermore, even when prospective matching is achieved successfully at the start of a study, subsequent hardware changes and software updates might still introduce acquisition effects in long multiyear prospective studies. In the future, software versions and coil updates could be considered as covariates of interest in RISH‐GLM to attempt limiting their effect on the acquired data.

When comparing scaling maps computed with RISH‐GLM using matched data sets to those computed with RISH (Figure 1), local relative differences up to 20% can be observed close to the WM‐GM and WM‐CSF interfaces. This is likely due to considering age and sex effects during estimation and suggests that such features may be locally relevant even in matched datasets. This observation is supported by the fact that relative differences between unmatched groups, shown in Figure 3, exhibit spatial patterns that are consistent with those of Figure 1, but with larger amplitude. Despite such local differences, when comparing FA and MD of matched groups (Figures 2 and S2), we did not observe any relevant group difference with both RISH and RISH‐GLM. However, our analysis clearly focused on the WM skeleton, whereas most differences between RISH and RISH‐GLM are located close to the WM‐GM interface. As such, future studies should investigate the potential of RISH and RISH‐GLM on more sophisticated analyses such as fiber tractography, where the transition of fibers between tissue types is crucial to achieve meaningful results.

It is important to acknowledge some limitations of this study. As explained in Section 2, RISH‐GLM is based on assumptions that might not always hold true. An important assumption is the existence of a linear relation between covariates of interest and RISH features, at least within the range of covariates used for training. In the case of age, for example, little is known about its exact relation to RISH features. Furthermore, RISH‐GLM has primarily been designed to account for systematic cross‐site biases, and its ability to capture non‐systematic effects leading to different subject ranking across sites^39^ remains unclear. Evaluating such aspects and validating the assumptions of RISH‐GLM throughout the lifespan will require dedicated studies, or an additional validation in cohorts including so‐called traveling heads (i.e., groups of subjects traveling across sites and scanned with highly controlled experimental conditions to ensure minimal variance of their measurements across sites,^13^, ^39^ ideally even considering confounders such as time of the day^40^). Nevertheless, we observe that RISH‐GLM is effective at recovering consistent relations between age and FA throughout the experiments of this work. Of note, the assumption of linearity between covariates and RISH features does not imply the need for linearity between covariates and derived metrics such as FA, given that RISH features are nonlinear descriptors of the diffusion signal. This is particularly relevant as previous studies^35^, ^41^ of diffusion tensor imaging metrics across the lifespan have demonstrated the existence of a quadratic—locally linear—relation between age and fractional anisotropy, for example. Another assumption of RISH‐GLM is that the effects of covariates is similar across training cohorts. As a consequence, training directly with non‐homogeneous groups, such as patients with different pathologies, is discouraged. In this study, we used data from a retrospective cohort of individuals that were initially marked as at risk for frontotemporal dementia, which might have introduced a selection bias toward effects not explicitly accounted for. Furthermore, we only focused on modeling the effect of age and sex and did not consider other relevant confounders such as education.

In conclusion, we have introduced a novel framework to learn cross‐site harmonization while accounting for covariates of interests; we demonstrated its effectiveness to learn harmonization between two age unmatched groups with an average age difference of over two decades.

## Supporting information


**Figure S1.** Percentage effect of age and sex estimated by rotational invariant spherical harmonics (RISH)–generalized linear model (GLM)[Matched]. Age effects are prevalent at the interface between cerebrospinal fluid and white/gray matter for $\vartheta_{0}$, and in the white matter for $\vartheta_{2}$ and $\vartheta_{4}$. The age effect map for $\vartheta_{6}$ appears to be dominated by noise effects, given the low anatomical contrast and the presence of clear stripes due to ghosting artifacts. Similar observations hold for sex effects.
**Figure S2.** *Top row*: Boxplots of average mean diffusivity (MD) values in the white‐matter skeleton per site, before harmonization (*first column*), after harmonization with rotational invariant spherical harmonics (RISH) (*middle column*), and with rotational invariant spherical harmonics–based generalized linear model (RISH‐GLM) (*last column*). *Second row*: Scatterplots of the same average MD values as a function of age. Harmonization with both RISH and RISH‐GLM was trained with group level–matched subjects.
**Figure S3.** Boxplots of average mean diffusivity (MD) values from two unmatched groups of healthy controls from Site 1 and Site 2, before harmonization and after harmonization, with rotational invariant spherical harmonics (RISH) and rotational invariant spherical harmonics–based generalized linear model (RISH‐GLM). Before harmonization, an unexpected negative relation between age and MD is observed. Harmonization with RISH removes any relation between age and MD. After harmonization with RISH‐GLM [Unmatched], a positive relation between age and MD is observed, as expected based on previous literature.
**Figure S4.** Scaling maps calculated between pairs of sites with rotational invariant spherical harmonics (RISH) and in one single step with rotational invariant spherical harmonics‐based generalized linear model (RISH‐GLM) on all three sites considered in Experiment 3.
**Figure S5.** Percentage effect of age and sex on rotational invariant spherical harmonics (RISH) features of different orders as determined with rotational invariant spherical harmonics‐based generalized linear model (RISH‐GLM).
**Figure S6.** Boxplots of average mean diffusivity (MD) values from two matched groups from Site 1 and Site 2 before harmonization, and after applying rotational invariant spherical harmonics (RISH) and rotational invariant spherical harmonics–based generalized linear model (RISH‐GLM) trained on unmatched data (Figure 5). No differences in average MD values are observed after harmonization with RISH‐GLM, as expected for two matched groups. The application of RISH‐GLM also allows us to reveal the same positive correlation between age and MD observed in the previous figures.

## Data Availability

The code used to perform RISH and RISH‐GLM harmonization is openly available at https://github.com/delucaal/RISH‐GLM.
