# Supplementary material for: Cross‐site harmonization of diffusion MRI data without matched training subjects
Source: Magn Reson Med. 2025 May 23;94(4):1750–62. doi: 10.1002/mrm.30575 (PMC12309894; doi:10.1002/mrm.30575)
Supplement: Supplementary file 1 — Figure S1. Percentage effect of age and sex estimated by rotational invariant spherical harmonics (RISH)–generalized linear model (GLM)[Matched]. Age effects are prevalent at the interface between cerebrospinal fluid and white/gray matter for ϑ0, and in the white matter for ϑ2 and ϑ4. The age effect map for ϑ6 appears to be dominated by noise effects, given the low anatomical contrast and the presence of clear stripes due to ghosting artifacts. Similar observations hold for sex effects. Figure S2. Top row: Boxplots of average mean diffusivity (MD) values in the white‐matter skeleton per site, before harmonization (first column), after harmonization with rotational invariant spherical harmonics (RISH) (middle column), and with rotational invariant spherical harmonics–based generalized linear model (RISH‐GLM) (last column). Second row: Scatterplots of the same average MD values as a function of age. Harmonization with both RISH and RISH‐GLM was trained with group level–matched subjects. Figure S3. Boxplots of average mean diffusivity (MD) values from two unmatched groups of healthy controls from Site 1 and Site 2, before harmonization and after harmonization, with rotational invariant spherical harmonics (RISH) and rotational invariant spherical harmonics–based generalized linear model (RISH‐GLM). Before harmonization, an unexpected negative relation between age and MD is observed. Harmonization with RISH removes any relation between age and MD. After harmonization with RISH‐GLM [Unmatched], a positive relation between age and MD is observed, as expected based on previous literature. Figure S4. Scaling maps calculated between pairs of sites with rotational invariant spherical harmonics (RISH) and in one single step with rotational invariant spherical harmonics‐based generalized linear model (RISH‐GLM) on all three sites considered in Experiment 3. Figure S5. Percentage effect of age and sex on rotational invariant spherical harmonics (RISH) features of different order [file MRM-94-1750-s001.docx]

# Supporting Information

#
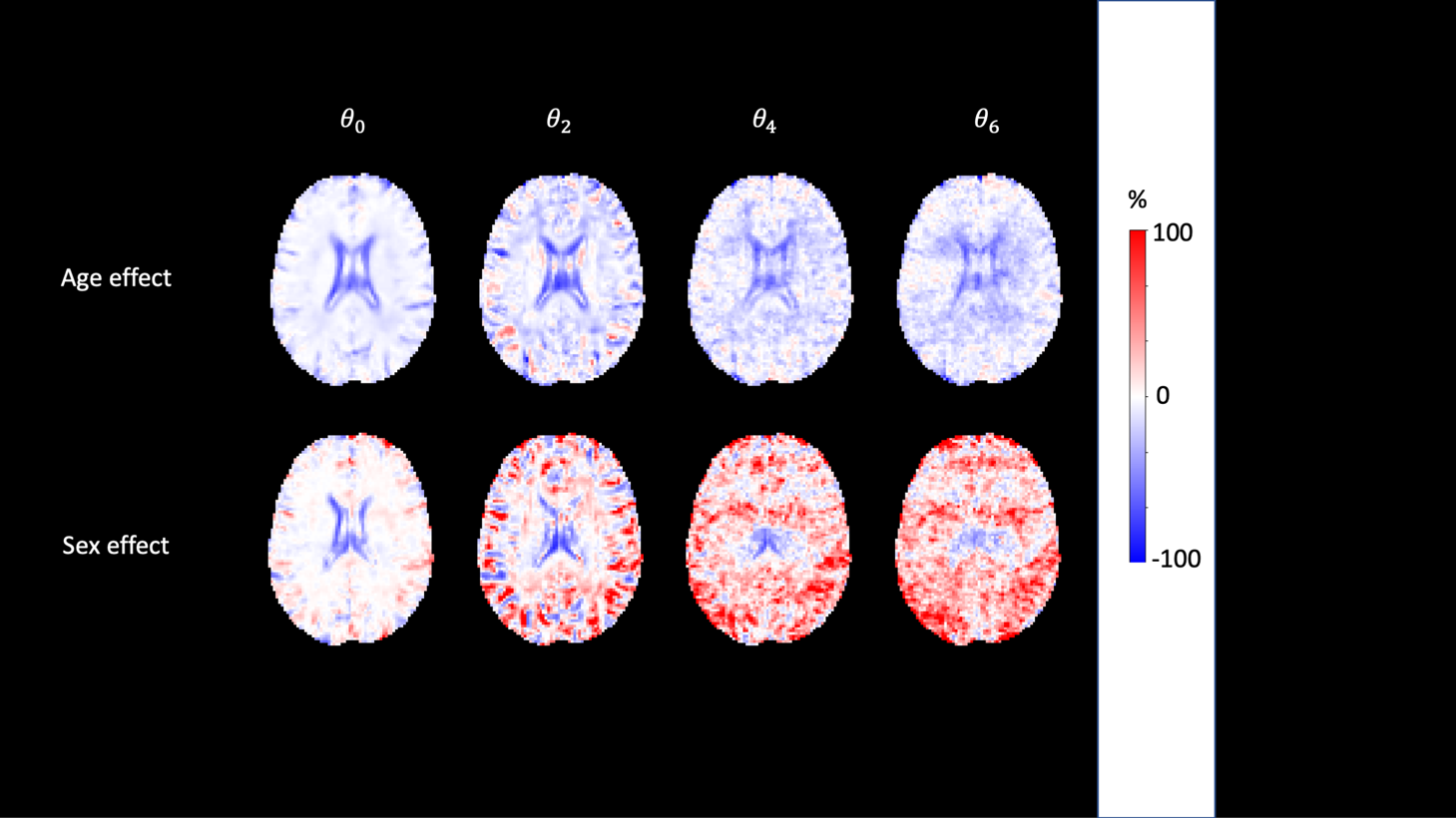


Figure S1: Percentage effect of age and sex estimated by RISH-GLM[Matched]. Age effects are prevalent at the interface between cerebrospinal fluid and white/grey matter for $\vartheta_{0}$, and in the white matter for $\vartheta_{2}$ and $\vartheta_{4}$. The age effect map for $\vartheta_{6}$ seems to be dominated by noise effects, given the low anatomical contrast, and the presence of clear stripes due to ghosting artefacts. Similar observations hold for sex effects.

#
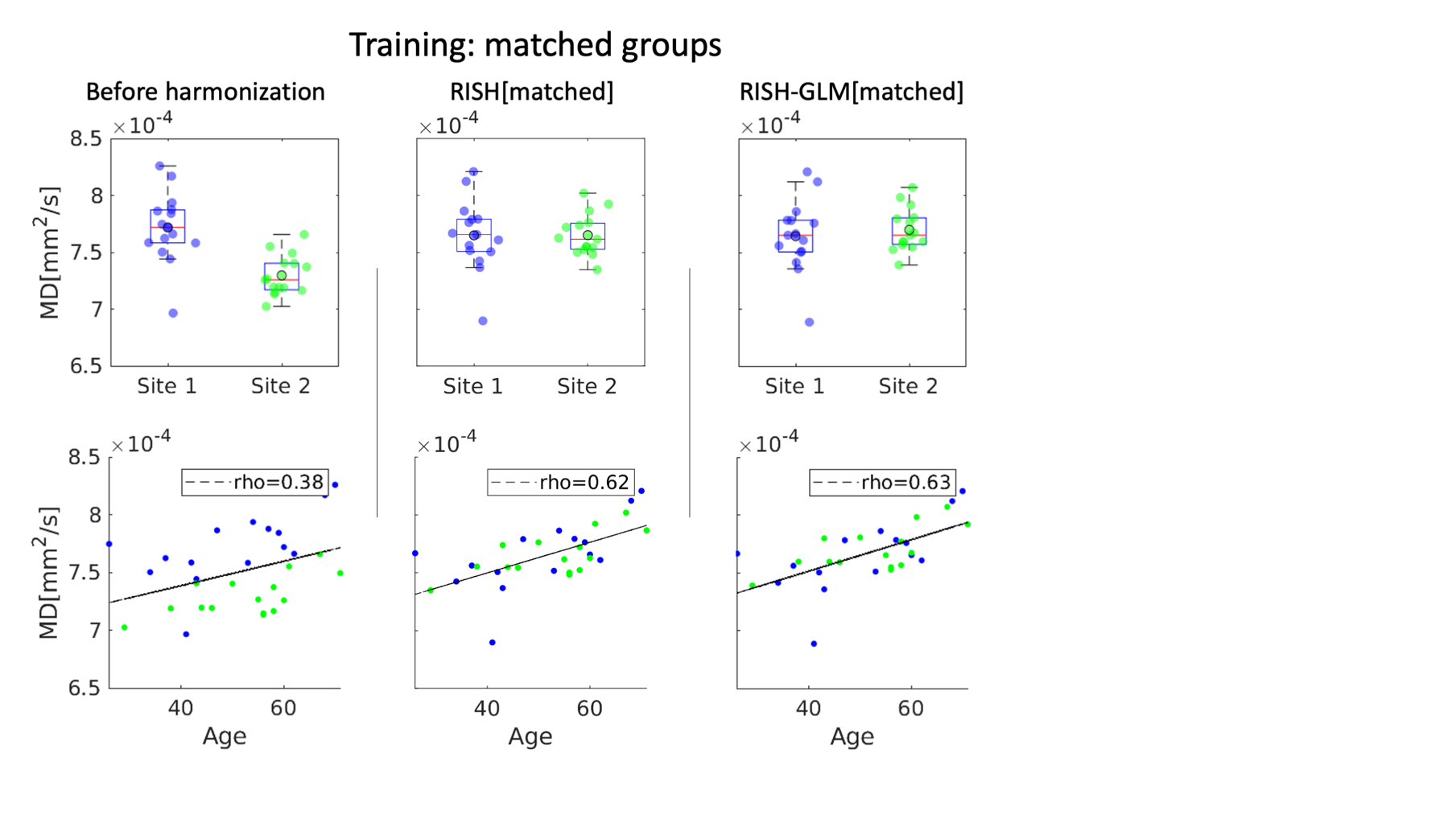


Figure S2: The top row shows boxplots of average MD values in the white matter skeleton per site, before harmonization (first column), after harmonization with RISH (middle column) and with RISH-GLM (last column). The second row shows scatterplots of the same average MD values as a function of age. Harmonization with both RISH and RISH-GLM was trained with group level-matched subjects.


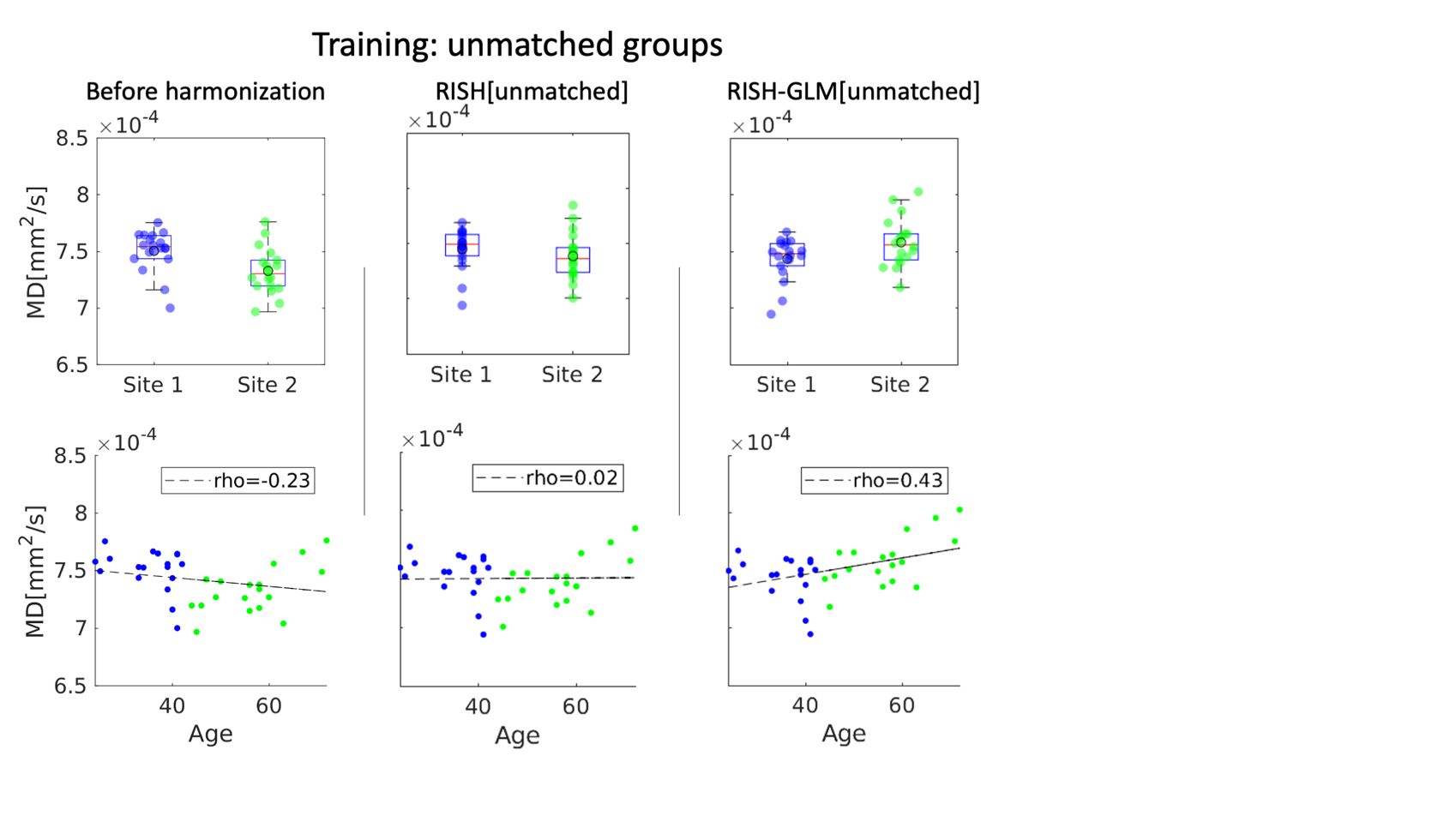


Figure S3: Boxplots of average MD values from two unmatched groups of healthy controls from Site1 and Site2, before harmonization, and after harmonization with RISH and RISH-GLM. Before harmonization an unexpected negative relation between age and MD is observed. Harmonization with RISH removes any relation between age and MD. After harmonization with RISH-GLM[Unmatched], a positive relation between age and MD is observed, as expected based on previous literature.


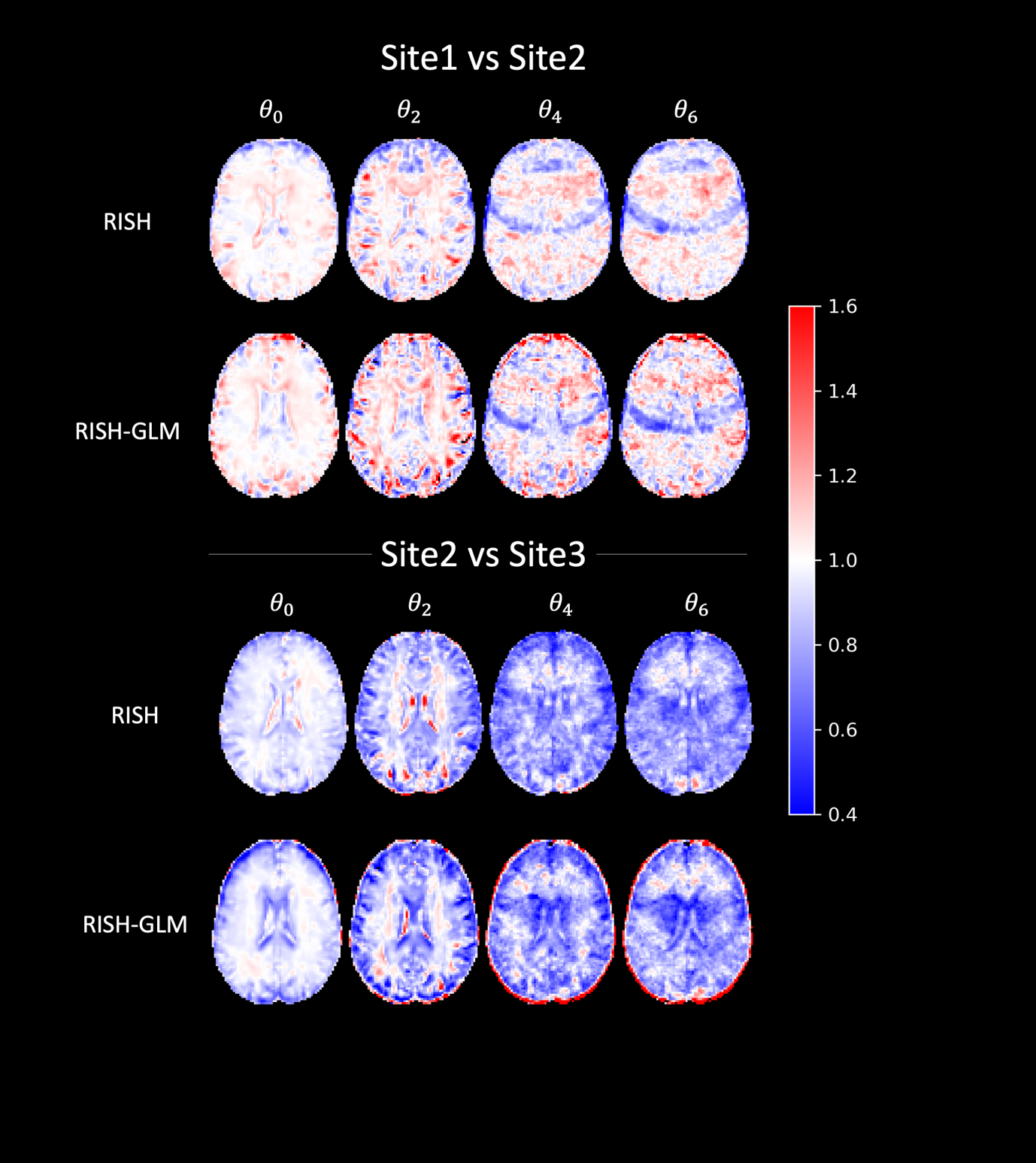


Figure S4: Scaling maps calculated between pairs of sites with RISH, and in one single step with RISH-GLM on all three sites considered in Experiment 3.


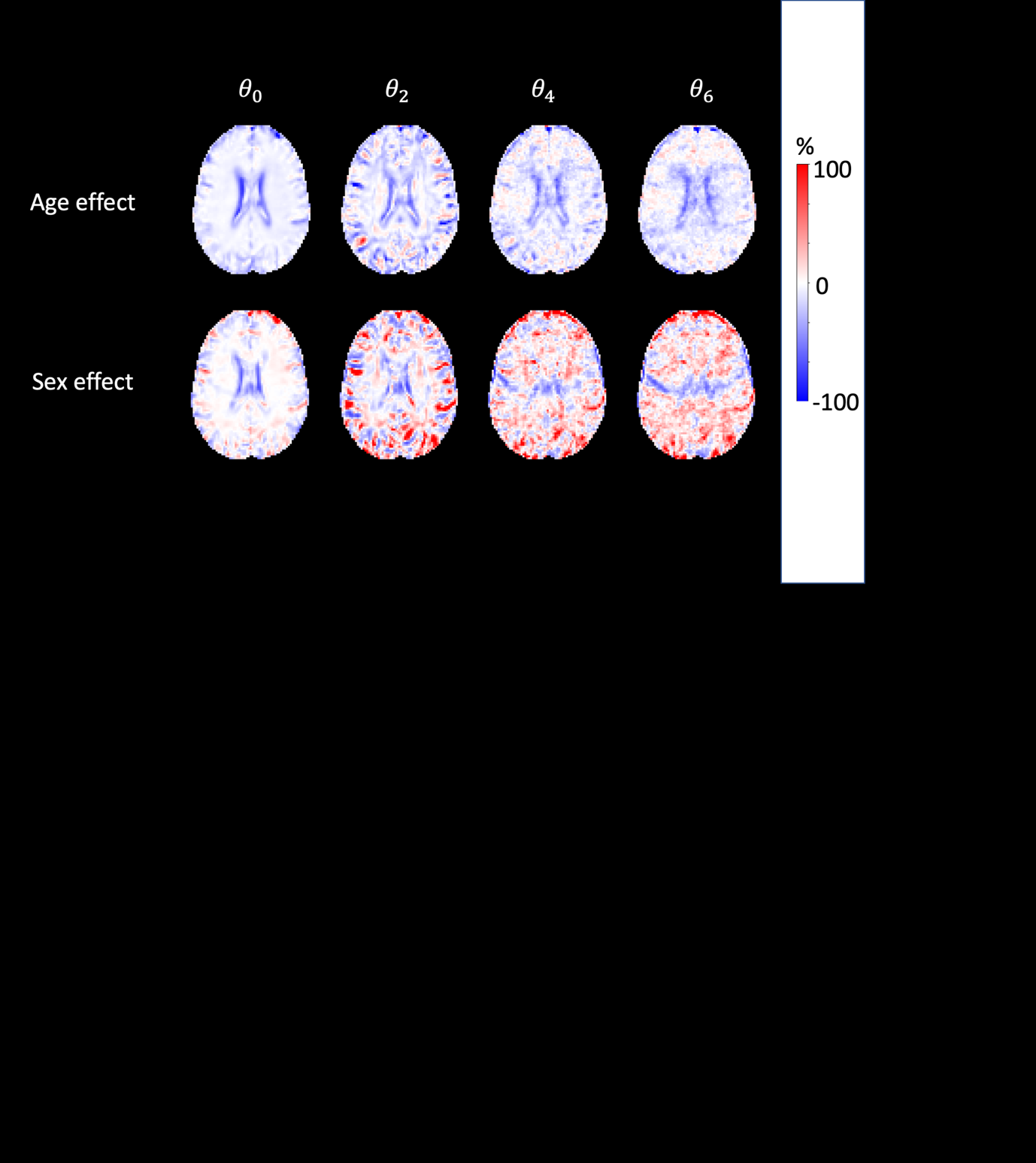


Figure S5: Percentage effect of age and sex on RISH features of different orders as determined with RISH-GLM.


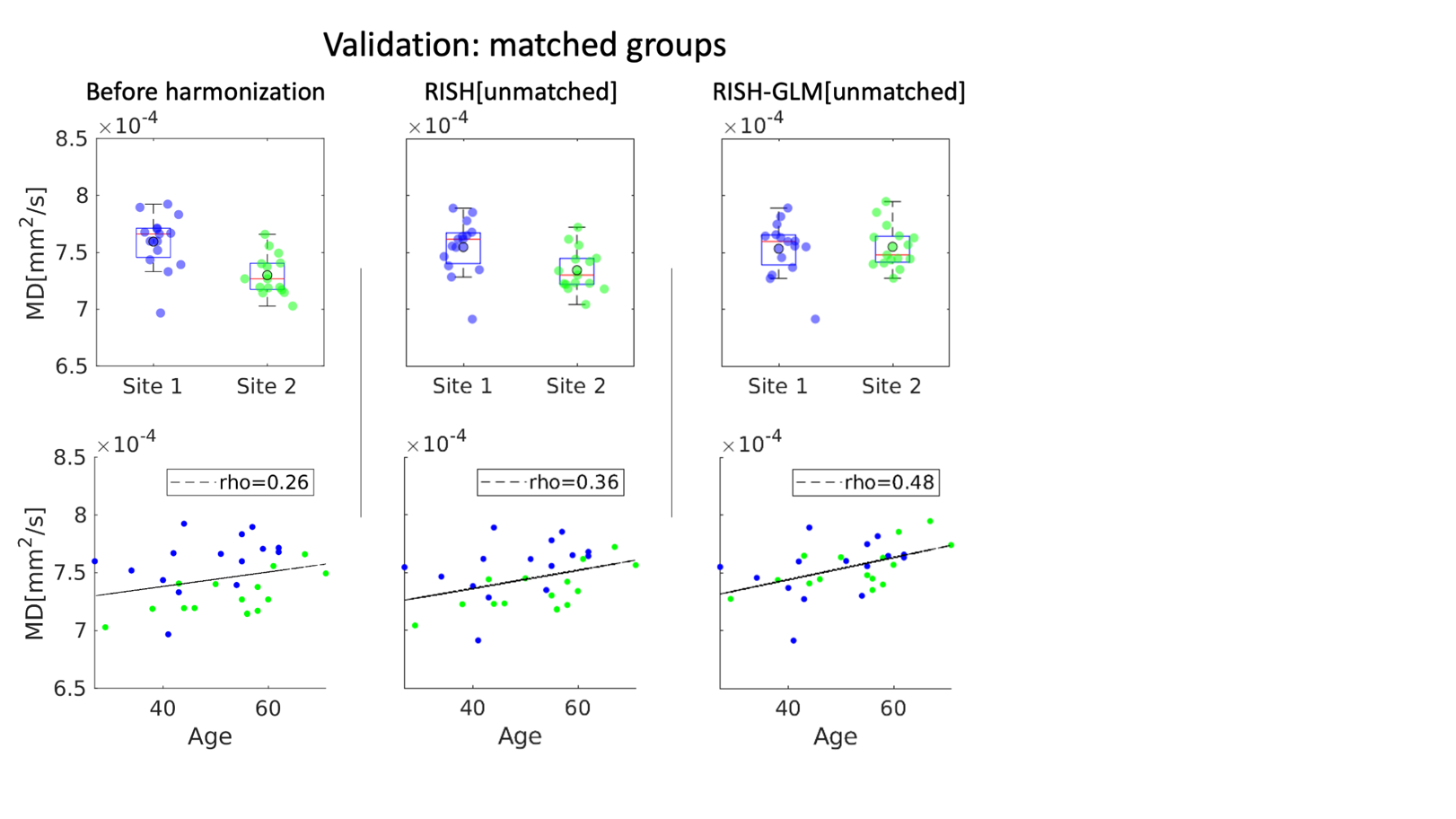


Figure S6: Boxplots of average MD values from two matched groups from Site1 and Site2 before harmonization, and after applying RISH and RISH-GLM trained on unmatched data (Figure 5). No differences in average MD values are observed after harmonization with RISH-GLM, as expected for two matched groups. The application of RISH-GLM also allows to reveal the same positive correlation between age and MD observed in the previous figures.
